# Myostatin Knockout Affects Mitochondrial Function by Inhibiting the AMPK/SIRT1/PGC1α Pathway in Skeletal Muscle

**DOI:** 10.3390/ijms232213703

**Published:** 2022-11-08

**Authors:** Mingjuan Gu, Zhuying Wei, Xueqiao Wang, Yang Gao, Dong Wang, Xuefei Liu, Chunling Bai, Guanghua Su, Lei Yang, Guangpeng Li

**Affiliations:** 1 State Key Laboratory of Reproductive Regulation and Breeding of Grassland Livestock, College of Life Science, Inner Mongolia University, Hohhot 010070, China; 2 College of Animal Science, Inner Mongolia Agricultural University, Hohhot 010018, China

**Keywords:** myostatin, CRISPR/Cas9, knockout, mitochondrial, skeletal muscle, AMPK/SIRT1/PGC-1α

## Abstract

**Simple Summary:**

Myostatin (*Mstn*) is a negative regulator of skeletal muscle mass, and its deletion leads to reduced mitochondrial function. However, the exact regulatory mechanism remains unclear. In this study, we used CRISPR/Cas9 to generate myostatin-knockout (*Mstn*-KO) mice via pronuclear microinjection. The skeletal muscle of *Mstn*-KO mice significantly increased, and the basal metabolic rate, muscle ATP synthesis, mitochondrial respiratory chain complex activity, tricarboxylic acid cycle (TCA), and thermogenesis decreased. In the muscle tissue of *Mstn*-KO mice, the expression of SIRT1 and pAMPK decreased, and the acetylation modification of PGC-1α increased. Furthermore, the treatment of isolated muscle cells from *Mstn*-KO and wild-type mice with AMPK activator (AICAR) and AMPK inhibitor (Compound C) found that Compound C down-regulated the expression of pAMPK and SIRT1 and the activity of citrate synthase (CS), isocitrate dehydrogenase (ICDHm) and α-ketoglutarate acid dehydrogenase (α-KGDH) similar to that of *Mstn*-KO. However, AICAR partially reversed the inhibitory effect of *Mstn*-KO on the expression of pAMPK and SIRT1 and activity of three enzymes. Thus, *Mstn*-KO affects mitochondrial function by inhibiting the AMPK/SIRT1/PGC1α signaling pathway.

**Abstract:**

Myostatin (*Mstn*) is a major negative regulator of skeletal muscle mass and initiates multiple metabolic changes. The deletion of the *Mstn* gene in mice leads to reduced mitochondrial functions. However, the underlying regulatory mechanisms remain unclear. In this study, we used CRISPR/Cas9 to generate myostatin-knockout (*Mstn*-KO) mice via pronuclear microinjection. *Mstn*-KO mice exhibited significantly larger skeletal muscles. Meanwhile, *Mstn* knockout regulated the organ weights of mice. Moreover, we found that *Mstn* knockout reduced the basal metabolic rate, muscle adenosine triphosphate (ATP) synthesis, activities of mitochondrial respiration chain complexes, tricarboxylic acid cycle (TCA) cycle, and thermogenesis. Mechanistically, expressions of silent information regulator 1 (SIRT1) and phosphorylated adenosine monophosphate-activated protein kinase (pAMPK) were down-regulated, while peroxisome proliferator-activated receptor γ coactivator-1α (PGC-1α) acetylation modification increased in the *Mstn*-KO mice. Skeletal muscle cells from *Mstn*-KO and WT were treated with AMPK activator 5-aminoimidazole-4-carboxamide riboside (AICAR), and the AMPK inhibitor Compound C, respectively. Compared with the wild-type (WT) group, Compound C treatment further down-regulated the expression or activity of pAMPK, SIRT1, citrate synthase (CS), isocitrate dehydrogenase (ICDHm), and α-ketoglutarate acid dehydrogenase (α-KGDH) in *Mstn*-KO mice, while *Mstn* knockout inhibited the AICAR activation effect. Therefore, *Mstn* knockout affects mitochondrial function by inhibiting the AMPK/SIRT1/PGC1α signaling pathway. The present study reveals a new mechanism for *Mstn* knockout in regulating energy homeostasis.

## 1. Introduction

Myostatin (*Mstn*) is a negative regulator of skeletal muscle mass [1]. *Mstn* knockout mice increased muscle fiber number (hyperplasia) and fiber size (hypertrophy) during development, resulting in a significant increase in muscle mass [1,2]. Consistently, naturally occurring mutations in *Mstn* gene generate similar muscle hypertrophy phenotypes in many different mammalian species, including cattle, sheep, dogs, and humans [3]. This gene has also been experimentally edited in different mammals including pigs [4,5], dogs [6], rabbits [7], goats [8,9], sheep [10], and cattle [11].

In addition, the lack of *Mstn* leads to the decline of ATP synthesis capacity [12,13]. Mitochondria are the main energy-converting organelles in eukaryotic cells, producing adenosine triphosphate (ATP) through the tricarboxylic acid cycle (TCA cycle) and oxidative phosphorylation (OXPHOS), which is the basic energy molecule of the cell [14]. The OXPHOS system is embedded in the inner mitochondrial membrane and consists of five complexes, namely complex I (CI), complex II (CII), complex III (CIII), complex IV (CIV), and complex V (CV). These enzymes catalyze the oxidation of biological substrates and the synthesis of ATP [15]. It is possible to directly or indirectly represent the respiratory function of mitochondria through the respiratory chain complex enzyme activity [16]. It has also been reported that the mitochondrial membrane potential (ΔΨm) represents the energy stored in the mitochondrial electric field for the conversion of ADP to ATP [17]. There are reports that mice with deletion of the *Mstn* gene exhibit a marked decrease in mitochondria content and disturbance in respiratory function [18,19]. However, the signaling mechanisms by which *Mstn* regulates mitochondria activity are still unknown.

AMPK (AMP-activated protein kinase), a serine/threonine protein kinase, plays a critical role in intracellular energy homeostasis and is essential for regulating mitochondrial function [20]. SIRT1 (silent information regulator 1) belongs to the sirtuins family of NAD+-dependent deacetylases and plays a role in regulating mitochondrial function [21]. PGC-1α (peroxisome proliferator-activated receptor gamma coactivator 1 alpha) is a transcriptional coactivator that regulates mitochondrial biogenesis and oxidative metabolism [22]. Interestingly, cross-talk among these energy-sensing factors regulates mitochondrial function. AMPK stimulates PGC1α activity by enhancing SIRT1-mediated PGC1α deacetylation [23]. AMPK regulates SIRT1 activity through NAD+content [23]. SIRT1 can directly interact and deacetylate PGC-1α [24]. PGC-1α function is associated with the regulation of large gene clusters that control oxidative phosphorylation and mitochondrial activity [25]. Canagliflozin (Cana) promoted mitochondrial biogenesis, mitochondrial oxidative phosphorylation, and thermogenesis via an AMPK–SIRT1–PGC1α pathway [26]. However, whether *Mstn* knockout regulates mitochondrial metabolism through the AMPK/SIRT1/PGC1alpha pathway was unknown.

In this study, we built *Mstn*-KO mice by CRISPR/Cas9 to explore the mechanism that how *Mstn* regulated mitochondrial activity. We found that basal metabolic rate, mitochondrial electron transport chain complexes, mitochondrial membrane potential, and TCA cycle were inhibited in *Mstn*-KO mice. Meanwhile, the expression of SIRT1 and pAMPK was down-regulated and increased PGC-1α acetylation in the *Mstn*-KO mice. These results indicated that *Mstn* knockout suppressed mitochondrial function probably by inhibiting the AMPK/SIRT1/PGC1alpha pathway.

## 2. Results

### 2.1. Generation of Mstn-KO Mice by CRISPR/Cas9 System 

**Figure 1:**
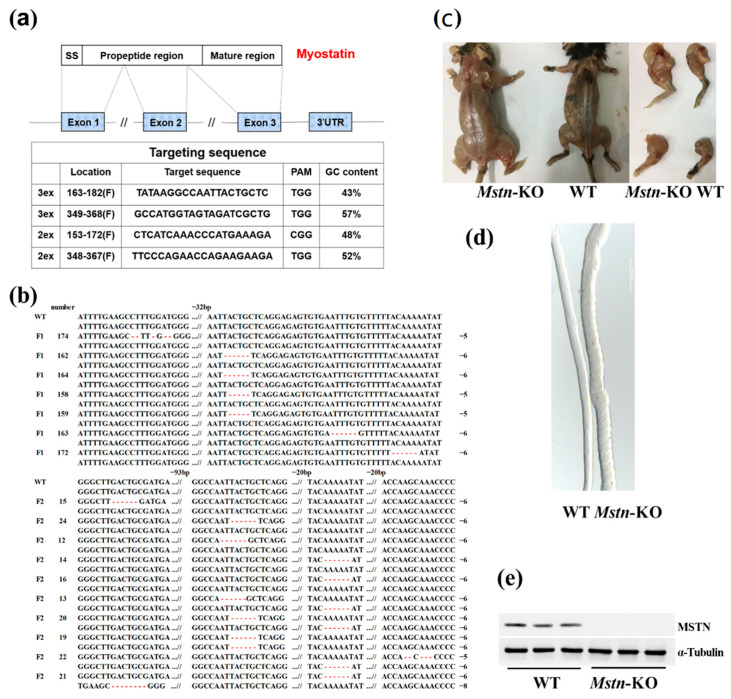
Production of myostatin knockout (*Mstn*-KO) mice mediated by CRISPR/Cas9 techniques. (**a**) gRNA sequence of the MSTN gene for CRISPR/Cas9. (**b**) Mutant *Mstn* genotypes of derived progenies, red indicates a missing base. (**c**) Representative images of muscles of *Mstn*-KO and WT mice. (**d**) Images of teased single muscle fibers for muscle fibers in *Mstn*-KO and WT mice. (**e**) Expression of MSTN protein in *Mstn*-KO and WT mice (*n* = 3).

To generate *Mstn*-KO mice by CRISPR/Cas9 techniques, we designed four guide RNAs to target exon 2 and exon 3 of the mouse MSTN gene, respectively (Figure 1a). Three transgenic founders were generated by pronuclear injection. To produce obvious phenotype *Mstn*-KO mice (F2), we mated only exon 3 deletion mutant F1 mice. A total of 10 mice (59%) among 17 F2 mice were identified as *Mstn* mutants, showing nine different genotypes with deletions ranging from 5 to 8 nt (Figure 1b). Phenotypic analysis showed that the *Mstn*-KO exhibited muscle hypertrophy in the skeletal muscle of *Mstn*-KO mice (Figure 1c). We isolated single muscle fibers from mice gastrocnemius and found that the *Mstn*-KO mice were significantly thicker than the WT mice (Figure 1d). Furthermore, MSTN protein expression was significantly decreased in the *Mstn*-KO mice compared with the WT mice (Figure 1e). 

### 2.2. Growth Performance and Phenotypic Traits

**Figure 2:**
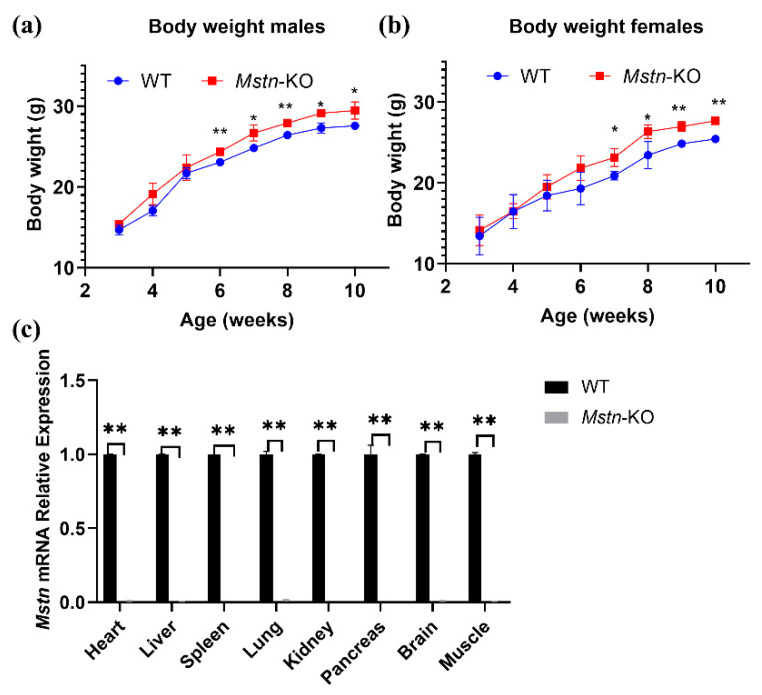
Comparison of growth performance between *Mstn*-KO and WT mice. (**a**) Comparison of body weights between *Mstn*-KO and WT male mice from 3 to 10 weeks. Body weight was slightly higher in *Mstn*-KO male mice than WT controls after 6 weeks (*n* = 3). (**b**) Comparison of body weights between *Mstn*-KO and WT female mice from 3 to 10 weeks. Body weight was slightly higher in *Mstn*-KO female mice than WT controls after 7 weeks (*n* = 3). (**c**) *Mstn* mRNA expression in organs of *Mstn*-KO and WT mice (*n* = 3). All data are presented as mean ± SD. * *p* < 0.05, ** *p* < 0.01; t-tests were used to calculate the p-values.

We compared and analyzed growth performances and phenotypic characteristics between *Mstn*-KO and WT mice (from 3 to 10 weeks). The average body weights of *Mstn*-KO and WT mice increased continuously from 3 to 10 weeks (Figure 2a,b). After 6 weeks, the average body weight of *Mstn*-KO male mice was significantly higher than WT mice (Figure 2a). However, the average body weight of *Mstn*-KO female mice was continuously higher than WT mice from 7 weeks (Figure 2b). Moreover, compared with the WT mice, the weights of the liver, spleen, lungs, thyroid, pancreas, brain, testis, and ovary were decreased by 0.55%, 33.51%, 10.01%, 0.26%, 14.51%, 24.23%, 30.79%, and 14.51%, respectively, in *Mstn*-KO. The heart and kidney weight of *Mstn*-KO mice was higher at 22.13% and 2.59% (Table 1). We investigated the expression of *Mstn* in the skeletal muscle and internal organs of WT and *Mstn*-KO mice using real-time quantitative PCR. *Mstn* mRNA expression in the heart, liver, lungs, kidneys, pancreas, brain, and muscle was significantly lower in *Mstn*-KO mice than in WT mice, while no signal was detected in the spleen (Figure 2c).

### 2.3. Effect of Mstn Knockout on Basal Metabolic Rate and Body Temperature

**Figure 3:**
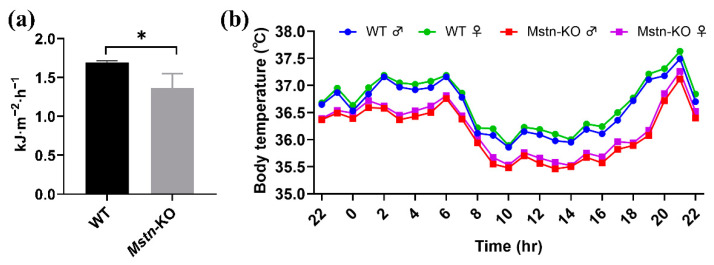
*Mstn* knockout decreases basal metabolic rate and body temperature. (**a**) Comparison of basal metabolic rate between *Mstn*-KO and WT mice (*n* = 3). (**b**) Body temperature in WT compared with *Mstn*-KO male and female mice (*n* = 1). All data are presented as mean ± SD. * *p* < 0.05; t-tests were used to calculate the *p*-values.

Next, we evaluated the basal metabolic rate (BMR) in *Mstn*-KO and WT mice. The levels of VO_2_, VCO_2_, and respiratory quotient (CO_2_ release/O_2_ consumption, RQ) of the resting state were examined in the groups. Compared to the WT mice, *Mstn*-KO mice consumed less O_2_ (2.40 ± 0.47 ml/min for *Mstn*-KO and 2.57 ± 0.25 ml/min for WT) and released less CO_2_ (1.65 ± 0.34 ml/min for *Mstn*-KO and 1.95 ± 0.26 ml/min for WT) (Table 2). RQ was reduced in *Mstn*-KO mice. *Mstn*-KO mice have a lower BMR (basal metabolic rate) compared with the WT mice (Figure 3a). In a resting state, the two major components of energy expenditure are the basal metabolic rate and maintaining body temperature. The body temperature of mice was monitored in real time over seven consecutive days. We found that body temperature was slightly lower in either male or female *Mstn*-KO mice compared to WT mice, and females had slightly higher body temperatures than males within the same group. (Figure 3b). However, the body temperature of these groups was maintained in the normal range. These results suggest that *Mstn* knockout reduced energy expenditure in a resting state.

### 2.4. Mstn Knockout Reduced Mitochondria Activity

**Figure 4:**
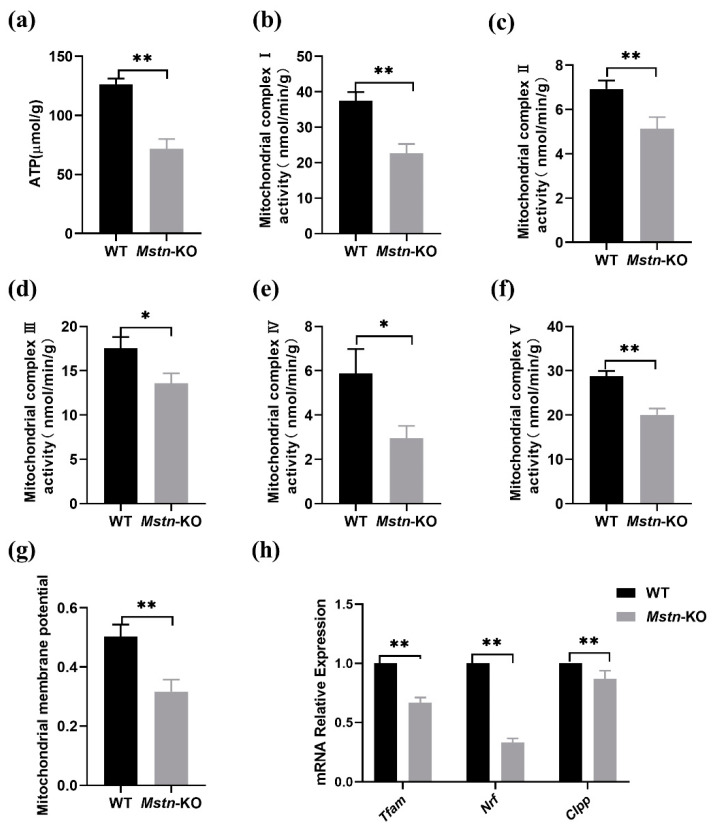
*Mstn* knockout reduced ATP content and mitochondria activity. (**a**) ATP content in *Mstn*-KO and WT mice muscle (*n* = 3). (**b**–**f**) Mitochondrial complexes I–V activity was analyzed by biochemical detection (*n* = 3). (**g**) Measurement of the mitochondrial membrane potential of *Mstn*-KO and WT mice (*n* = 3). (**h**) mRNA levels of mitochondrial activity gene by qPCR (*n* = 3). All data are presented as mean ± SD. * *p* < 0.05, ** *p* < 0.01; t-tests were used to calculate the *p*-values.

It is well-established that mitochondria are the center of cellular energy metabolism. The mitochondrial TCA cycle and the electron transport chain are the two main components that determine mitochondrial energy metabolism. Firstly, we measured the total ATP content of muscle tissues. The results were that ATP synthesis was significantly decreased in the *Mstn*-KO mice (Figure 4a). To further explore the effects of *Mstn* on the main processes of energy metabolism, we examined the individual activities of mitochondrial electron transport chain complexes I-V. The data showed that the activities of complexes I to V were reduced 0.6-fold, 0.74-fold, 0.77-fold, 0.53-fold, and 0.7-fold in *Mstn*-KO mice, respectively (Figure 4b–f). Furthermore, *Mstn* knockout decreased mitochondrial membrane potential compared with the WT mice (Figure 4g). We further investigated the mRNA levels of the mitochondrial activity gene by qPCR. Lower transcript levels of Tfam, Nrf, and CIpp genes were found in *Mstn*-KO mice (Figure 4h).

### 2.5. Mstn Knockout Inhibited the TCA Cycle

**Figure 5:**
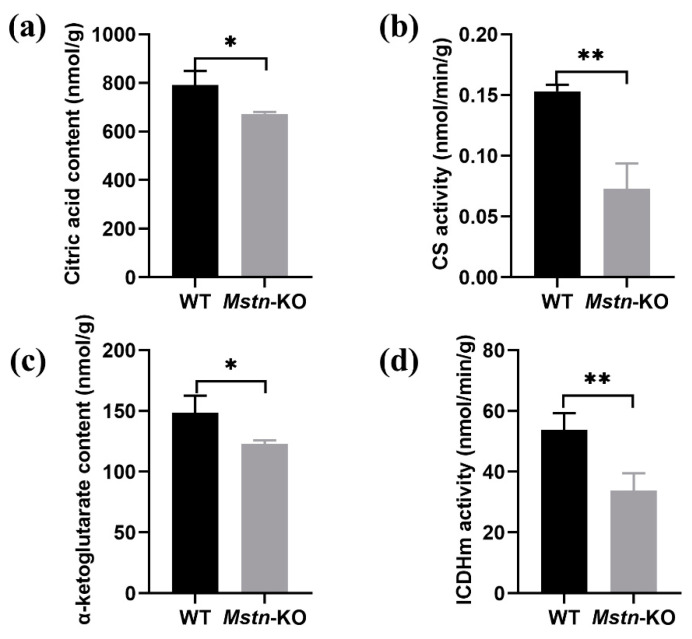
Key enzymes and metabolites in the tricarboxylic acid (TCA) cycle. (**a**) Citrate acid content of the initial step product in the TCA cycle (*n* = 3). (**b**) Citrate synthase activity in *Mstn*-KO and WT mice (*n* = 3). (**c**) α-ketoglutarate content in *Mstn*-KO and WT mice (*n* = 3). (**d**) Isocitrate dehydrogenase activity in *Mstn*-KO and WT mice (*n* = 3). All data are presented as mean ± SD. * *p* < 0.05, ** *p* < 0.01; t-tests were used to calculate the *p*-values.

We further examined key enzymes and metabolites in the TCA cycle. Citrate synthase and citrate acids are enzymes and a product of the initial step in the TCA cycle. As shown in Figure 5a,b, citrate content and citrate synthase activity were reduced in the *Mstn*-KO mice compared to the WT mice. In addition, isocitrate dehydrogenase activity and α-ketoglutarate content were decreased in the *Mstn*-KO mice (Figure 5c,d). Isocitrate dehydrogenase converts isocitrate to α-ketoglutarate in the TCA cycle. These results indicated that *Mstn* knockout decreased mitochondrial function whether electron transport chain or TCA cycle. 

### 2.6. Mstn Knockout Inhibited AMPK/SIRT1/PGC1alpha Pathway

**Figure 6:**
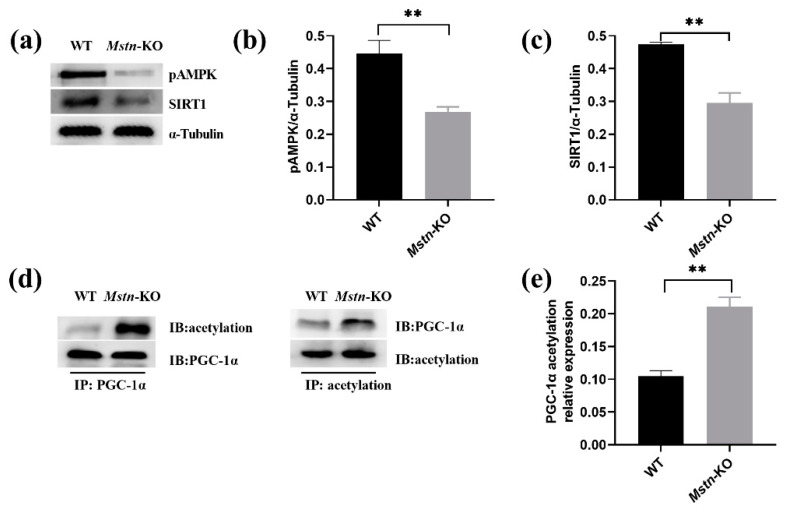
*Mstn* knockout inhibited the AMPK/SIRT1/PGC1alpha pathway. (**a**) The expression of SIRT1 and pAMPK at the protein level in *Mstn*-KO and WT mice. (**b**) Gray intensity analysis of pAMPK/α-Tubulin (*n* = 3). (**c**) Gray intensity analysis of SIRT1/α-Tubulin (*n* = 3). (**d**) Acetylation level of PGC1α protein by co-immunoprecipitation in *Mstn*-KO and WT mice. (**e**) Gray intensity analysis of acetylation level of PGC1α (*n* = 3). All data are presented as mean ± SD. ** *p* < 0.01; t-tests were used to calculate the *p*-values.

Previous studies have demonstrated that AMPK regulates mitochondrial function [27]. Thus, we next determined whether the phosphorylation level AMPK changes in *Mstn*-KO mice skeletal muscle. As expected, compared to the WT mice, the expression of pAMPK was significantly downregulated (Figure 6a,b). We further examined the expression of SIRT1 of the downstream molecule of AMPK by Western blot. *Mstn*-KO mice had significantly decreased levels of SIRT1 (Figure 6a,c). Moreover, to investigate the acetylation level of PGC1α protein in *Mstn*-KO and WT mice, we detected the acetylated PGC1α protein in *Mstn*-KO and WT mice. Since no commercial acetylation antibody of PGC1α protein was available, the pan acetyllysine antibody was used to assess the acetylation level of PGC1α by IP analysis. Briefly, PGC1α was pulled down with the anti-PGC1α antibody, and an IP/Western blot assay was carried out to analyze the acetylation of PGC1α using the previously reported method [28,29]. *Mstn* knockout resulted in PGC1α acetylation increase, suggesting that PGC-1α activity was decreased (Figure 6d,e). This finding supports previous findings that PGC-1α expression is subject to auto-regulation in collaboration with SIRT1, which activates PGC-1α through deacetylation. Taken together, these findings show that *Mstn* knockout inhibited the AMPK/SIRT1/PGC1alpha pathway.

### 2.7. Expression of pAMPK and SIRT1 following Treatment with AICAR and Compound C

**Figure 7:**
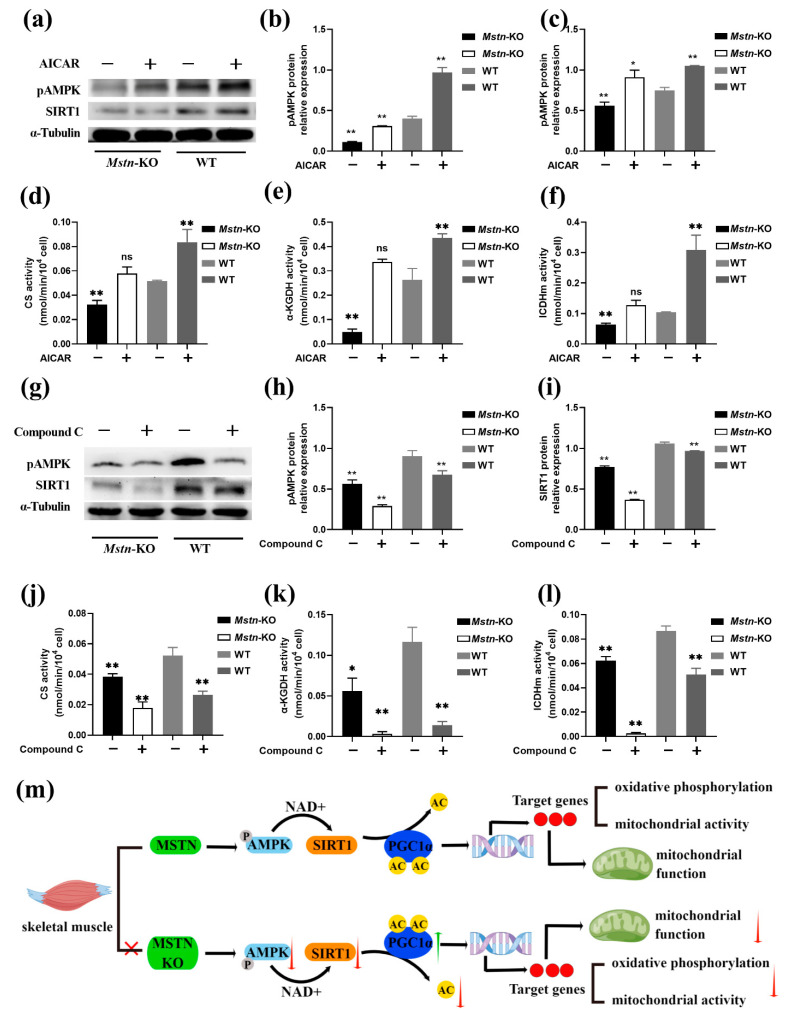
Expression of pAMPK and SIRT1 following treatment with AICAR and Compound C. (**a**) The expression of SIRT1 and pAMPK with AICAR treatment *Mstn*-KO and WT cells. (**b**,**c**) Quantitative analysis showing the expression of pAMPK and SIRT1 following treatment with AICAR (*n* = 3). (**d**) Citrate synthase (CS) activity with AICAR treatment *Mstn*-KO and WT cells (*n* = 3). (**e**) α-ketoglutarate acid dehydrogenase (α-KGDH) activity with AICAR treatment *Mstn*-KO and WT cells (*n* = 3). (**f**) Isocitrate dehydrogenase (ICDHm) activity with AICAR treatment *Mstn*-KO and WT cells (*n* = 3). (**g**) The expression of SIRT1 and pAMPK with Compound C treatment *Mstn*-KO and WT cells. (**h**,**i**) Quantitative analysis showing the expression of pAMPK and SIRT1 following treatment with Compound C (*n* = 3). (**j**) Citrate synthase (CS) activity with Compound C treatment *Mstn*-KO and WT cells (*n* = 3). (**k**) α-ketoglutarate acid dehydrogenase (α-KGDH) activity with Compound C treatment *Mstn*-KO and WT cells (*n* = 3). (**l**) Isocitrate dehydrogenase (ICDHm) activity with Compound C treatment *Mstn*-KO and WT cells (*n* = 3). (**m**) Pattern of MSTN knockdown affecting mitochondrial function through the AMPK/SIRT1/PGC1α pathway. All data are presented as mean ± SD. ns, non-significant *p* > 0.05; * *p* < 0.05; ** *p* < 0.01; t-tests were used to calculate the *p*-values.

To further explore the relationship between MSTN and the AMPK/SIRT1/PGC1α pathway in skeletal muscle mitochondrial function, cells of *Mstn*-KO and WT mice were treated with AMPK activator AICAR and the AMPK inhibitor Compound C. AICAR is an AMP analog. Similar to AMP, AICAR binds to the γ subunit of AMPK, allosterically activates the enzyme, stimulates phosphorylation at Thr^172^ by liver kinase B1 (LKB1), and protects against pThr^172^ dephosphorylation [30]. Compound C is an ATP-competitive inhibitor and binds to the highly conserved active site of AMPK [31]. SIRT1 and pAMPK protein expression and activity of citrate synthase (CS), isocitrate dehydrogenase (ICDHm), and α-ketoglutarate acid dehydrogenase (α-KGDH) were determined by Western blotting analysis and biochemical detection methods, respectively. AICAR-treated cells exhibited increased pAMPK and SIRT1 expression compared to AICAR-untreated cells (Figure 7a–c). The expressions of pAMPK and SIRT1 proteins were decreased in MT cells compared with WT cells (Figure 7a–c). Meanwhile, the activity of CS, ICDHm, and α-KGDH in the treated and untreated cells obtained similar results (Figure 7d–f). The AICAR effects were inhibited by the *Mstn* knockout (Figure 7a–f). Furthermore, *Mstn*-KO cells showed dramatically decreased pAMPK and SIRT1 protein levels and activity of the three enzymes compared to WT cells, and the same results were also obtained in the treated group compared with the untreated group (Figure 7g–l). The SIRT1 and pAMPK expression and activity of the three enzymes of *Mstn*-KO cells were reduced further in response to compound c stimulation (Figure 7g–l). These results suggest that *Mstn* knockout reduces mitochondrial function by inhibiting AMPK–SIRT1 signaling.

## 3. Discussion

*Mstn* is a potent inhibitor of skeletal muscle mass [32]. *Mstn* knockout animals all showed a skeletal muscle hypertrophy phenotype [7,33,34,35,36,37,38]. The *Mstn*-knockout mice we obtained also showed a hypertrophic phenotype. In addition to the characteristic effects on the skeletal muscle, *Mstn* knockout can affect other organs. Currently, the weight of organs has previously been reported on cattle, mice, and piglets of *Mstn* deficiency. At 15 or 20 months of age, the weights of the heart, liver, spleen, and lungs of Charolais double muscle cattle decreased by 20%, 20%, 30%, and 10%, respectively [39]. Moreover, at 4, 8, and 12 weeks of age, the kidneys and liver of *Mstn*-deficient mice were lighter than those of WT mice, while the weight of the heart and lungs were similar [40]. In MSTN-KO piglets, the weights of the heart, liver, lungs, kidneys, and stomach were decreased by 21.4%, 21.3%, 29.8%, 16.7%, and 20.0% relative to body weight, respectively [41]. In the current study, the weights of the liver, spleen, lungs, thyroid, pancreas, brain, testis, and ovary were decreased by 0.55%, 33.51%, 10.01%, 0.26%, 14.51%, 24.23%, 30.79%, and 14.51%, respectively, whereas the heart and kidney weight of *Mstn*-KO mice was higher in 22.13% and 2.59%. We reasoned that *Mstn* might exert a different effect on organ weight in different species.

*Mstn* knockout is closely related to skeletal muscle metabolism [42,43]. Several studies have reported that *Mstn* knockout decreases ATP production during exercise [12,44]. Moreover, Li et al. found that knockout *Mstn* in loach significantly decreased ATP synthesis by directly measuring the total ATP content of loach muscle tissue [13]. In this study, we demonstrated that *Mstn* knockout muscle decreased ATP synthesis in the resting state of *Mstn*-KO mice. Interestingly, we also found that the basal metabolic rate and body temperature were significantly decreased in *Mstn*-KO mice correlating with the reduced ATP synthesis capacity. Indeed, mitochondria produce most of the ATP in cells [45]. Our present study confirmed that mitochondrial electron transport chain complexes, mitochondrial membrane potential, and TCA cycle were reduced in *Mstn*-KO mice muscle. These results further reveal that *Mstn* knockout impact mitochondrial function, as studied previously in *Mstn* KO animals [13,18,19].

AMPK, SIRT1 and PGC1α are all involved in regulating mitochondrial function [20,21,22]. According to Price and his colleagues [46], resveratrol improves mitochondrial biogenesis and function by activating SIRT1. SIRT1 activates PGC1α by deacetylation [47]. Melatonin prevents mitochondrial fission through the SIRT1–PGC1α pathway [48]. Meanwhile, salidroside may treat diabetic nephropathy in mice through SIRT1–PGC1α mediated mitochondrial biogenesis [49]. In addition, AMPK regulates SIRT1 activity by modulating intracellular NAD^+^ levels and thereby influencing PGC1α deacetylation [50]. We have found, for the first time, that *Mstn* knockout down-regulated the expressions of SIRT1 and pAMPK, enhancing PGC-1α acetylation. Skeletal muscle cells from *Mstn*-KO and WT were treated with AMPK activators AICAR and the AMPK inhibitor Compound C, respectively. Compared with WT mice, Compound C treatment further down-regulated the expression of pAMPK and SIRT1 expression and activity of CS, ICDHm, and α-KGDH in *Mstn*-KO mice, while *Mstn* knockout inhibited the AICAR activation effect. Therefore, *Mstn* knockout inhibited mitochondrial function via the AMPK/SIRT1/PGC1α signaling pathway.

## 4. Materials and Methods

### 4.1. Mstn-KO Mouse Production and Validation

The *Mstn*-KO mice were generated by pronuclear microinjection. The sgRNA oligos were synthesized and cloned into the pCas-Guide-EF1α-GFP plasmid downstream at the *BamHⅠ* and *BsmBⅠ* restriction sites to generate the pCas-Guide-EF1α-GFP-sgRNA recombinant plasmid. The positive clones were confirmed by Sanger sequencing. The purified transgene was microinjected into the male pronuclei of fertilized eggs from superovulated female mice and transferred to recipient pseudopregnant females. The mouse genotypes were determined by PCR-based assays; the primers used for genotyping are listed in Table 3.

### 4.2. Body Temperature Measurements

The body temperature of the animals was measured daily by a subcutaneously located temperature chip.

### 4.3. Metabolic Measurements 

Mice were individually housed in the metabolic cages (Oxylet) and acclimatized for 24 h before recording. Their 24 h oxygen consumption (VO_2_), carbon dioxide production (VCO_2_), and respiratory quotient (RQ) were measured every hour for 3 min in each cage. Mice were maintained on their normal diet or water throughout the detection process.

### 4.4. Characterization and Analysis of Organs

Healthy mice from each group (*Mstn*-KO and WT) were euthanized and tissues were collected for experimental purposes. The organs analyzed were the spleen, brain, lungs, pancreas, heart, liver, kidney, thyroid, testicle, and ovary. Body weight and organ weight were calculated.

### 4.5. Western Blot 

The total protein was extracted from the muscle and cells of *Mstn*-KO and WT mice according to our previously reported method [51]. Proteins were detected using primary antibodies, including anti-MSTN (Abcam, Cambridge, MA, USA, ab201954), anti-pAMPK (Abcam, Cambridge, MA, USA, ab133448), anti-PGC-1α (Santa Cruz Biotechnology, Santa Cruz, CA, USA, sc-518025), and anti-acetyllysine (PTM BIO, Hang Zhou, China, PTM-101).

### 4.6. Real-Time PCR 

Real-time PCR was performed referring to our previous reported [51]. Primer sequences were as tabulated in Table 3.

### 4.7. Biochemical Detection

Enzyme activities and metabolites were assayed using an ATP content assay kit (ATP-1-Y), mitochondrial electron transport chain complexes I-V assay kit (FHTA-1-Y, FHTB-1-Y, FHTC-1-Y, FHTD-1-Y and FHTE-1-Y), citrate synthase activity assay kit (CS-1-Y), citrate acid content assay kit (CA-1-W), isocitrate dehydrogenase activity assay kit (ICDHM-1-Y), α-ketoglutarate acid dehydrogenase activity assay kit (KGDH-1-Y), and α-ketoglutarate content assay kit (KGA-4-Q) according to the manufacturer’s protocols from Comin (Su Zhou, China). The optical densities were measured using a microplate reader (Thermo, Waltham, MA, USA).

### 4.8. Co-Immunoprecipitation

Lysates of mice skeletal muscle tissue generated under the addition of proteinase inhibit cocktail Complete Mini (Thermo, Waltham, MA, USA) and phosphatase inhibitor cocktail PhosSTOP (Thermo, Waltham, MA, USA). The total protein of the lysates was measured by the Pierce BCA Protein Assay Kit (Thermo, Waltham, MA, USA). Co-immunoprecipitation (co-IP) was completed using the Thermo Scientific Pierce co-IP kit (#26149) following the manufacturer’s protocol. Ten micrograms of the antibody were incubated with the delivered resin and covalently coupled. The antibody-coupled resin was incubated with 200 µL of the whole mice skeletal muscle protein lysates overnight at 4 °C, respectively. The resin was washed, and the protein complexes bound to the antibody were eluted. Subsequent Western blot analyses were performed as described before.

### 4.9. Cell Culture and Treatment

Primary mouse skeletal muscle cell was cultured using a described method before [52]. Cells were treated with 1 mM AICAR (Selleck, Shanghai, China, S1802) or 5 µM Compound C (Selleck, Shang Hai, China, S7306).

### 4.10. Statistical Analysis

Comparisons between two observations in the same subjects were assessed by Student’s paired t-test. Results were expressed as the mean ± standard deviation (SD). The *p*-value of less than 0.05 was accepted as statistical significance.

## 5. Conclusions

This study demonstrated that *Mstn* knockout decrease basal metabolic rate, body temperature, and mitochondrial activity in skeletal muscle. The probable mechanism is that *Mstn* knockout suppressed mitochondrial function via inhibiting the AMPK/SIRT1/PGC1α signaling pathway (Figure 7m).

## Figures and Tables

**Table 1:** Organ weight of *Mstn*-KO and WT mice.

	Heart	Liver	Spleen	Lung	Kidney	Thyroid	Pancreas	Brain	Testis	Ovary
KO (g)	0.20 ± 0.03	1.71 ± 0.45	0.07 ± 0.01	0.20 ± 0.01	0.48 ± 0.00	0.21 ± 0.01	0.26 ± 0.04	0.39 ± 0.01	0.17 ± 0.01	0.02 ± 0.00
WT (g)	0.14 ± 0.00	1.47 ± 0.22	0.09 ± 0.01	0.19 ± 0.01	0.40 ± 0.00	0.18 ± 0.01	0.26 ± 0.01	0.44 ± 0.01	0.21 ± 0.01	0.02 ± 0.00
%	22.13	−0.55	−33.51	−10.01	2.59	−0.26	−14.51	−24.23	−30.79	−14.51

All values are presented as mean ± SD (*n* = 6). KO, *Mstn*-KO mice; WT, WT mice.

**Table 2:** The basic energy metabolism of *Mstn*-KO and WT mice.

	O_2_	CO_2_	RQ	Weight/g	BMR
KO	2.40 ± 0.47	1.65 ± 0.34	0.69 ± 0.02	27.23 ± 0.58	1.36
WT	2.57 ± 0.25	1.95 ± 0.26	0.76 ± 0.14	22.15 ± 0.84	1.69

All values are presented as mean ± SD (*n* = 3). KO, *Mstn*-KO mice; WT, WT mice.

**Table 3:** Primers used for real-time PCR and genotyping PCR.

Gene Name	Sense (5′ to 3′)	Anti-Sense (5′ to 3′)
*Tfam*	TGAAGCTTGTAAATGAGGCTTGGA	CGGATCGTTTCACACTTCGAC
*Nrf*	TTTGGCGCAGCACCTTTG	GAGGCGGCAGCTCTGAATTAAC
*CIpp*	CACACCAAGCAGAGCCTACA	TCCAAGATGCCAAACTCTTG
*GAPDH*	AAATGGTGAAGGTCGGTGTGAAC	CAACAATCTCCACTTTGCCACTG
*Mstn-2ex*	CAACAAAGTAGTAAAAGCCCAA	ACTTTGTCTGGCTTATGAGCAT
*Mstn-3ex*	AGTCAAGGTGACAGACACACCC	GTGCTTGAATTCACAGTTTCGA

## Data Availability

The data presented in this study are available on request from the corresponding author.
